# Performance of 16s rDNA Primer Pairs in the Study of Rhizosphere and Endosphere Bacterial Microbiomes in Metabarcoding Studies

**DOI:** 10.3389/fmicb.2016.00650

**Published:** 2016-05-13

**Authors:** Bram Beckers, Michiel Op De Beeck, Sofie Thijs, Sascha Truyens, Nele Weyens, Wout Boerjan, Jaco Vangronsveld

**Affiliations:** ^1^Centre for Environmental Sciences, Hasselt UniversityDiepenbeek, Belgium; ^2^Department of Plant Systems Biology, Flanders Institute for Biotechnology (VIB)Gent, Belgium; ^3^Department of Plant Biotechnology and Bioinformatics, Ghent UniversityGent, Belgium

**Keywords:** 16S rDNA metabarcoding, 454 pyrosequencing, plant microbiome, chloroplast DNA, endophytes

## Abstract

Next-generation sequencing technologies have revolutionized the methods for studying microbial ecology by enabling high-resolution community profiling. However, the use of these technologies in unraveling the plant microbiome remains challenging. Many bacterial 16S rDNA primer pairs also exhibit high affinity for non-target DNA such as plastid (mostly chloroplast) DNA and mitochondrial DNA. Therefore, we experimentally tested a series of commonly used primers for the analysis of plant-associated bacterial communities using 454 pyrosequencing. We evaluated the performance of all selected primer pairs in the study of the bacterial microbiomes present in the rhizosphere soil, root, stem and leaf endosphere of field-grown poplar trees (*Populus tremula* × *Populus alba*) based on (a) co-amplification of non-target DNA, (b) low amplification efficiency for pure chloroplast DNA (real-time PCR), (c) high retrieval of bacterial 16S rDNA, (d) high operational taxonomic unit (OTU) richness and Inverse Simpson diversity and (e) taxonomic assignment of reads. Results indicate that experimental evaluation of primers provide valuable information that could contribute in the selection of suitable primer pairs for 16S rDNA metabarcoding studies in plant-microbiota research. Furthermore, we show that primer pair 799F-1391R outperforms all other primer pairs in our study in the elimination of non-target DNA and retrieval of bacterial OTUs.

## Introduction

The development and implementation of next-generation sequencing technologies (NGS) and their corresponding bioinformatics tools have revolutionized the methods for studying microbial ecology by enabling high-resolution community profiling (Margulies et al., 2005; Sogin et al., 2006; Parameswaran et al., 2007; Shendure and Ji, 2008; Metzker, 2010; Caporaso et al., 2011). DNA sequencing of 16S rDNA genes or gene fragments have fuelled large microbial community studies in humans [Andersson et al., 2008; Qin et al., 2010; HMPC (Human Microbiome Project Consortium), 2012], the gut microfauna of insects (Sudakaran et al., 2012; Hansen and Moran, 2014), different natural habitats (Andrew et al., 2012; DeLeon-Rodriguez et al., 2013; Hamdan et al., 2013) as well as plant microbiome studies (Gottel et al., 2011; Mendes et al., 2011; Bulgarelli et al., 2012; Lundberg et al., 2012; Peiffer et al., 2013; Shakya et al., 2013; Edwards et al., 2015). Currently, the most used next generation sequencing platforms in the profiling of microbiomes are the 454 pyrosequencing technology (Margulies et al., 2005) and the Illumina MiSeq and HiSeq systems (Caporaso et al., 2011, 2012). 454 pyrosequencing has continously demonstrated its effectiveness in describing microbial communities by enabling highly multiplexed sequencing of short hypervariable 16S rDNA regions (Gottel et al., 2011; Lundberg et al., 2012; Bulgarelli et al., 2013; Shakya et al., 2013). In recent years, the Illumina HiSeq and MiSeq systems have also earned their place in high-throughput 16S rDNA sequencing and have even surpassed 454 pyrosequencing in terms of read quantity and quality (Caporaso et al., 2012).

For both technologies, several studies have evaluated their technical aspects such as error profiles (Kunin et al., 2010; Balzer et al., 2011; Gilles et al., 2011; Nakamura et al., 2011; Schirmer et al., 2015), platform-specific effects on the observed microbial communities (Claesson et al., 2010; Luo et al., 2012; Nelson et al., 2014; Tremblay et al., 2015) as well as biases introduced by primer specificity with *in silico* studies (Klindworth et al., 2013) and *in vivo* studies (Aird et al., 2011; Berry et al., 2011; Pinto and Raskin, 2012; Kennedy et al., 2014). Another important aspect within microbial ecology and 16S rDNA community profiling with NGS techniques is the occurence of contaminating sequences, which are routinously co-extracted during DNA extraction from various biotic samples. In numerous research areas, NGS-based approaches are susceptible to contamination with undesired sequences (non-target DNA) such as in the diagnosis of symptomatic infections (microbial DNA contamination; Strong et al., 2014), in malaria clinical sequencing (human DNA contamination) (Oyola et al., 2013), and food web analysis (Pompanon et al., 2012). More specifically within microbial ecology, in the study of interorganismal association such as the human microbiome [HMPC (Human Microbiome Project Consortium), 2012], plant-microbiome associations (Lundberg et al., 2012; Bodenhausen et al., 2013; Bulgarelli et al., 2013; Ghyselinck et al., 2013) and insect-microbiome studies (Sudakaran et al., 2012; Hansen and Moran, 2014) sequences from an organellar origin (e.g., mitochondria and/or chloroplast DNA) represent a major source of contamination.

This is of particular interest in plant-microbiome research since plants house eukaryotic cells, prokaryotic cells, and eukaryotic plant organelles with a prokaryotic lineage (mitochondria and chloroplast/plastids) (Dyall et al., 2004; Raven, 1970). The number of mitochondria and chloroplasts vary depending on the plant species, cell type and age of the tissue but can be as high as 10,000 chloroplast DNA copies in tobacco leaf cells (Shaver et al., 2006). The homology between bacterial 16S rDNA, chloroplast DNA, and mitochondrial DNA leads to significant challenges in the selection of appropriate primer pairs to study plant-microbe interactions (Ghyselinck et al., 2013). Currently, three general methods exist to reduce the impact of these contamining sequences: (a) adaptation of existing DNA extraction protocols to reduce co-extraction of organellar DNA (Lutz et al., 2011) or post-extraction separation of host DNA from microbial DNA based on differences in CpG methylation density (Feehery et al., 2013), (b) the development of blocking primers to block and/or reduce amplification of sequences originating from a eukaryotic host such as peptide nucleic acid-mediated PCR clamping (Lundberg et al., 2013) and suicide polymerase endonuclease restriction (SuPER) (Green and Minz, 2005), and (c) the use of specific mismatch primers during PCR amplification (Chelius and Triplett, 2001; Sakai et al., 2004).

The preferred or most utilized technique is the use specific mismatch primers, which amplify bacterial 16S rDNA sequences while simultaneously avoiding the amplification of chloroplast DNA sequences. Chelius and Triplett (2001) developed the first mismatch primer (799F), with a primer design which centered around two base pair mismatches at positions 798–799 and two additional base pair mismatches at positions 783 and 784 in the chloroplast DNA. Primer 799F has been used with varying success in several plant systems (Bulgarelli et al., 2012; Bodenhausen et al., 2013; Shade et al., 2013). Further, Sakai et al. (2004) modified primer 799F into primer 783Rabc, in an attempt to access the hypervariable regions V3-V4 of the bacterial 16S rDNA genes in the study of the rhizobacterial communities of wheat and spinach. Indeed hypervariable regions V3 and V4 have been the preferred target of the 16S rDNA in studying soil and rhizosphere assemblages and databases are more exhaustive for these regions (Klindworth et al., 2013). Furthermore, Rastogi et al. (2010) used primer 783Rabc to develop a PCR-based method to determine the degree of chloroplast and mitochondrial contamination in DNA samples from plant environments.

However, the experimental performance of these mismatch primers (and their potential to reduce co-amplification of non-target DNA) in different plant compartments with low chloroplast/plastid input (rhizosphere soil) and higher chloroplast/plastid input (endosphere compartments) has not been evaluated. Although, *in silico* analyses provide valuable technical information and indicate the theoretical optimal performance of primer pairs, they fail to capture the true experimental potential and are expected to result in an incomplete picture of how primers will perform during PCR amplification (Op De Beeck et al., 2014). Therefore, experimental evaluation of the amplification efficiency and robustness of selected primer pairs in plant-bacteria interaction studies is essential to assess their behavior in these specific conditions.

For this reason, we experimentally tested a set of commonly used primers for the analysis of plant-associated bacterial communities using 454 pyrosequencing (Table 1). We tested all selected primer pairs in the study of plant-associated bacterial communities in rhizosphere, roots, stems, and leaves of hybrid poplar trees (*Populus tremula* × *P. alba*). The different amounts of plastid DNA content of these plant compartments, ranging from virtually no plastid content (rhizosphere soil) to very high plastid (chloroplast) content (leaves) allows us to evaluate the performance of the selected primer sets in specific conditions.

**Table 1:** **Summary of primers used in the current study**.

**Primer pairs**	**Primer sequence (5′−3′)**	**A**	**B**	**C**	**D**	**References**
799F	AACMGGATTAGATACCCKG	79.7	0.29	4	V5-V6-V7	Chelius and Triplett, 2001
1391R	GACGGGCGGTGWGTRCA	84.6	1.44	0		Walker and Pace, 2007
967F	CAACGCGAAGAACCTTACC	80.9	0.34	0	V6-V7	Sogin et al., 2006
1391R	GACGGGCGGTGWGTRCA	84.6	1.44	0		Walker and Pace, 2007
799F	AACMGGATTAGATACCCKG	79.7	0.29	4	V5-V6-V7	Chelius and Triplett, 2001
1193R	ACGTCATCCCCACCTTCC	78.1	0.20	0		Bodenhausen et al., 2013
341F	CCTACGGGNGGCWGCAG	91.2	0.05	0	V3-V4	Klindworth et al., 2013
785R	GACTACHVGGGTATCT AATCC	86.2	0.09	0		Klindworth et al., 2013
68F	TNANACATGCAAGTCGRRCG	72.5	0.60	0	V1-V4	McAllister et al., 2011
783Rabc	CTACC^*^AGGGTATCTAATCC^*^TG	70.9	5.05	3		Sakai et al., 2004
68F	TNANACATGCAAGTCGRRCG	72.5	0.60	0	V1-V3	McAllister et al., 2011
518R	WTTACCGCGGCTGCTG G	87.6	0.09	0		Lee et al., 2010
341F	CCTACGGGNGGCWGCAG	91.2	0.05	0	V3-V4	Klindworth et al., 2013
783Rabc	CTACC^*^AGGGTATCTAATCC^*^TG	70.9	5.05	3		Sakai et al., 2004

*Primers are indicated as forward (F) or reverse (R)*.

* *Primer 783Rabc is a primer mix (Sakai et al., 2004)*:

*(a) 5′-CTACCAGGGTATCTAATCCTG-3′*,

*(b) 5′-CTACCGGGGTATCTAATCCCG-3′*,

*(c) 5′- CTACCCGGGTATCTAATCCGG-3′*.

*(A) Primer coverage (%) for Bacteria using Silva (Quast et al., 2013) and probeBase (Loy et al., 2007), (B) Primer Score tested with PrimerProspector 1.0.1 (Walters et al., 2011) (Table S1), (C) Number of mismatches with poplar chloroplast DNA, (D) Hypervariable region of the 16S rRNA operon targeted by primer pairs*.

## Materials and methods

### Study site description and sampling

A poplar (*Populus tremula* × *Populus alba*, cv “717-1-B4,” female clones) field trial located in Ghent, Belgium (property of VIB) was selected to acquire samples for this study (Custers, 2009). The field trial was established in April 2009 with a density of 15,000 trees per hectare and an inter-plant distance of 0.75 m (Van Acker et al., 2014). Briefly, poplar trees were sampled after ~3.5 years of growth in October 2012. We collected samples from rhizosphere soil, roots, stems, and leaves of three biologically independent poplar individuals. Per poplar individual, we collected (a) 10 g of roots at a depth of 5–10 cm below ground in 50 mL plastic tubes, (b) one complete offshoot for the stem and leaf samples. To standardize and maximize reproducibility of stem samples, several small “cores” with bark (5–7 cores, each about 1 cm) were collected from each offshoot. Finally all leaves of each offshoot were gathered and placed in sealed plastic bags for transportation.

### Processing of samples

Poplar root samples were shaken for 10 min on a shaking platform (100 rpm) and soil particles dislodged from the roots were collected as rhizosphere soil. Rhizosphere soil was sieved using a 2 mm sieve for homogenization and removal of residual roots and debris. Subsequently the samples were stored at −80°C until DNA was extracted.

Epiphytes (microbes living on the plant surface) were removed from all plant samples (roots, stems, and leaves) via surface-sterilization under aseptic conditions. Samples were sequentially washed with (a) sterile Millipore water (30 s), (b) followed by immersion in 70% (v/v) ethanol (2 min), (c) sodium hypochlorite solution (2.5% active Cl^−^, 5 min) supplemented with 0.1% Tween 80, (d) 70% (v/v) ethanol (30 s) and finalized by rinsing the samples five times with sterile Millipore water. Finally plant samples (~5 g of each compartment per sample) were homogenized by (a) portioning the samples into small fragments using a sterile scalpel and (b) macerating them in sterile 10 mM phosphate saline (PBS) buffer (130 mM NaCl, 7 mM Na_2_HPO_4_, 3 mM NaH_2_PO_4_, pH 7.4) using a Polytron PR1200 mixer (Kinematica A6) in four cycles of 2 min with cooling of the mixer on ice between cycles to reduce heating of the samples. Finally, quadruplicate aliquots of each sample (1.5 mL) of the homogenized plant material (root, stem, and leaf) were stored for all poplar individuals at −80°C until DNA was extracted.

### DNA extraction

DNA from rhizosphere, roots, stems, and leaves (further denoted as “plant compartments”) was extracted in quadruplicate from three biologically independent poplar individuals to minimize DNA extraction bias (Feinstein et al., 2009; Op De Beeck et al., 2014). In total, DNA was extracted from 48 samples (3 poplar individuals × 4 plant compartments × 4 quadruplicate extractions per sample).

Approximately 250 mg of rhizosphere soil was used for each individual DNA extraction. DNA extraction was performed with the Power Soil DNA Isolation Kit following the protocol provided by the manufacturer (MoBio, Carlsbad, CA, USA). For the plant compartments (roots, stems, leaves), aliquots (1.5 mL) of homogenized plant material were first centrifuged (13.400 rpm, 30 min) to collect all cells. Supernatants were discarded and DNA extractions were performed on pelleted plant material. After optimalization of the DNA extraction kit (Figure S1), DNA was extracted from the plant samples using the Invisorb Spin Plant Mini Kit according to the manufacturer's protocol (Stratec Biomedical AG, Birkenfeld, Germany).

### *In silico* evaluation of primer pairs

To select suitable primer pairs from all available 16S rDNA primers, several parameters (Table 1):

- Primer coverage and phylum spectrum of all 16S rDNA primers were queried using data from Silva (Klindworth et al., 2013; Quast et al., 2013) and probeBase (Loy et al., 2007).
- To evaluate the primer-to-target 3′ mismatches, primers were tested with PrimerProspector 1.0.1 (Walters et al., 2011) against the Greengenes database (gg_13_5.fasta) containing 1,262,986 sequences (DeSantis et al., 2006). This database was curated using mothur (version 1.34.3) to remove sequences containing ambiguous bases and sequences with homopolymers longer than eight bases. The curated database contained 946,815 16S rDNA sequences with an average read length of 1399.4 basepairs. All primer tests were performed as described by Walters et al. (2011) using standard settings. Primer scores were calculated based on the following formula: weighted score = non-3′ mismatches × 0.40+3′ mismatches × 1.00 + non-3′ gaps × 1.00+3′ gaps × 3.00. An additional penalty score of 3.00 was assigned if the final 3′ base of a primer had a mismatch with its target sequence.
- The presence of mismatches with poplar chloroplast DNA was evaluated by downloading the full chloroplast genome of *Populus alba* from NCBI and blasting each primer using Fast PCR (Kalendar et al., 2014).
- Overall coverage of the mostly used hypervariable regions of the 16S rRNA operon and the resulting amplicon length.

### PCR amplification and 454 pyrosequencing

For the PCR amplification, we selected seven different primer pairs to evaluate their performance in metabarcoding studies for rhizospheric and endophytic bacteria (Table 1). Primer pairs covered all hypervariable regions from V1 until V7 of the 16S rDNA gene and included, amongst others, primer 799F (5′-AACMGGATTAGATACCCKG-3′) (Chelius and Triplett, 2001) and primer 783Rabc (5′-CTACC*AGGGTATCTAATCC*TG; Sakai et al., 2004), which theoretically minimize chloroplast contamination by providing considerable mismatches with the poplar plastid DNA (3–4 mismatches). All primers and their sequences are listed in Table 1. Except for primer pair 68F-518R, all forward primers were fused to the Roche 454 pyrosequencing adaptor A and a sample-specific 10 bp barcode (multiplex identifiers, MIDs) and all reverse primers were fused to adaptor B (Roche Applied Science, Mannheim, Germany). For primer pair 68F-518R, the reverse primer was fused to adaptor A and the forward primer was fused to adaptor B.

DNA samples (*n* = 48) were individually amplified using a Techne TC-5000 thermocycler (Bibby Scientific Limited, Staffordshire, UK) with the seven different primer pairs. Since the concentration of bacterial DNA in comparison with the plant DNA is low, we chose a nested PCR strategy to amplify all samples and thereby minimize the formation of primer dimers. A first round of PCR amplification was conducted using primers without the Roche 454 pyrosequencing adaptors and sample-specific barcode. Each 25 μl PCR reaction contained ~10 ng of DNA and was carried out using the FastStart High Fidelity PCR System (Roche Applied Science, Mannheim, Germany). Each reaction contained 2.75 μl FastStart 10 x reaction buffer, 1.8 mM MgCl_2_, 0.2 mM dNTP mix, 0.4 μM of each primer, and 2 U FastStart HiFi polymerase. Cycling conditions included: initial denaturation at 94°C for 3 min, followed by 35 cycles of denaturation at 94°C for 1 min, annealing at 53°C for 1 min, and extension at 72°C during 1 min; a final extension phase was performed at 72°C during 10 min. PCR amplicon pools were cleared from residual primers and primer dimers by separating the PCR products on a 1.5% agarose gel. Bacterial amplicons were excised from the gels using the QIAQuick gel extraction kit (Qiagen Benelux N.V., Venlo, The Netherlands). Mitochondrial products produced by primers 799F-1391R and 799F-1193R of respectively 1000 and 800 bp were also eliminated via the gel purification (Figure S2). Following the first round of PCR amplification and gel-purification of the PCR products, a second round of PCR amplification was carried out for all seven primer pairs with the Roche 454 pyrosequencing adaptors and the sample specific barcodes. Amplicon length of sequences produced by primer pairs 799F-1391R and 68F-783Rabc was reduced by amplifying the samples with 967F-1391R and 68F-518R in the second round. PCR cycling conditions were identical as previously described, with the exception of the number of PCR cycles, which was lowered to 25.

Subsequently, quadruplicate PCR amplicon pools from the corresponding samples were pooled together to end up with 12 samples from four different compartments (rhizosphere, root, stem, leaf) of three biologically independent poplar individuals. PCR amplicon pools were purified to remove residual PCR primers and primer dimers using the QIAquick PCR purification kit (Qiagen Benelux B.V., Venlo, The Netherlands). Following purification, the quality of the amplicon pools was evaluated using an Agilent 2100 Bioanalyzer system (Agilent Technologies, Diegem, Belgium) according to the manufacturer's protocol. Finally, purified amplicon libraries were quantified with the Quant-iT PicoGreen dsDNA Assay Kit (Invitrogen, Carlsbad, CA, USA) and a Fluostar Omega plate reader (BMG Labtech, Ortenberg, Germany) and pooled in equimolar concentrations. The resulting seven amplicon pools (one for each primer pair), each of them containing 12 samples, were sequenced on one eighth of a Pico Titer Plate on a Roche Genome Sequencer FLX+ using Titanium chemistry (Roche Applied Science, Mannheim, Germany) by LGC Genomics (Berlin, Germany). Total amplicon pools consisted of 26 samples, including 14 samples related to another study.

### Sequence processing

Sequencing generated seven individual Standard Flowgram Format (SFF) files, which were analyzed separately using the software package mothur (version 1.34.3) following the Standard Operating Procedure outlined in http://www.mothur.org/wiki/Schloss_SOP (Schloss et al., 2009). Briefly, sequencing errors were reduced by denoising (shhh.flows, mothur implementation of Amplicon Noise algorithm) and quality trimming, which removed reads shorter than 200 bases, reads with homopolymers longer than eight bases and reads containing ambiguous bases. Unique sequences were identified, whilst archiving the abundance data of the unique sequences, and aligned using align.seqs with the SILVA reference alignment (Release 119) (Pruesse et al., 2007). Within the unique sequences, chimeric sequences were identified using the Uchime tool (*de novo* chimera detection) (Edgar et al., 2011) followed by their removal from the dataset. Taxonomic classification of the sequences were done using a cut-off of 80%. Sequences matching “Chloroplast” and “Mitochondria” were identified using classify.seqs and abundance data of these sequences were used to compare the performance of all primer pairs (Table 2). Subsequently these sequences were removed from the data set. Finally, genus-level OTUs (Operational Taxonomic Unit) were defined based on a 97% sequence similarity level. Complete parametrical evaluation was conducted with primer pairs 799F-1391R, 799F-1193R, and 341F-783Rabc based on low co-amplification of non-target DNA and high retrieval of bacterial reads. Because these selected primer pairs resulted in differential amounts of reads per sample, the number of reads per sample were rarefied to 417 reads per sample. Samples, for which fewer than 417 reads were obtained, were removed from the data set. Only for primer pair 341F-783Rabc, we removed 3 samples, all belonging to the stem compartment. Rarefaction curves were assembled based on 10,000 permutations and intra-sample richness, diversity, and Good's coverage estimates which were calculated in mothur (version 1.34.3) based on 10,000 iterations.

**Table 2:** **Quality metrics of pyrosequencing analysis, co-amplification of non-target DNA, and amplification of bacterial rDNA reads**.

**A. Total reads**	**799F-1391R**	**967F-1391R**	**799F-1193R**	**341F-785R**	**68F-783Rabc**	**68F-518R**	**341F-783Rabc**
Rhizosphere soil	2235±165	2550±673	956±285	1961±119	3346±454	1519±217	2196 ± 317
Root	2728±74	2577±56	1943±129	2916±438	2484±155	3488±532	2548 ± 403
Stem	2811±117	2502±159	2456±486	2199±350	2068±384	3412±632	1386 ± 18
Leaf	2665±100	2402±231	2410±197	2621±134	1961±64	3257±367	1678 ± 81
Read length before QC	405±96	401±101	364±105	392±105	348±139	349±105	361 ± 129
Read length after QC	207±4	208±4	217±5	233±5	222±5	200±4	205 ± 4
**NORMALIZATION TO 1000 READS**							
**B. Chloroplast DNA**	**799F-1391R**	**967F-1391R**	**799F-1193R**	**341F-785R**	**68F-783Rabc**	**68F-518R**	**341F-783Rabc**
Rhizosphere soil	0 ^a^	0.2±0.3 (< 0.1)^a^	0 ^a^	1±2 (0.1)^a^	0 ^a^	0 ^a^	0.2±0.3 (< 0.1)^a^
Root	0 ^a^	786±79 (79)^b^	0 ^a^	863±54 (86)^b^	736±90 (74)^b^	975±8 (97)^c^	270±87 (26)^d^
Stem	2 ± 3(0.2)^a^	997±3 (99)^b^	0 ^a^	962±1 (96)^b^	993±4 (99)^b^	998±1 (99)^b^	804±36 (80)^c^
Leaf	0 ^a^	907±35 (91)^b^	0 ^a^	910±29 (91)^b^	894±12 (89)^b^	985±4 (98)^c^	518±71 (52)^d^
**C. Mitochondrial DNA**	**799F-1391R**	**967F-1391R**	**799F-1193R**	**341F-785R**	**68F-783Rabc**	**68F-518R**	**341F-783Rabc**
Rhizosphere soil	0 ^a^	0 ^a^	0.5±0.5 (< 0.1)^a^	0 ^a^	0 ^a^	0 ^a^	0 ^a^
Root	0 ^a^	0 ^a^	9±1 (1)^b^	45±17 (5) ^c^	15±5 (1) ^b^	4±1 (0.5)^b^	136±17 (14)^d^
Stem	0 ^a^	0 ^a^	19±11 (2)^b^	35±1 (4)^b^	6±3 (0.5)^a^	1±1 (0.1)^a^	173±25 (17)^c^
Leaf	0 ^a^	0 ^a^	11±2.5 (1)^b^	69±16 (7)^c^	20±13 (2)^b^	6±3 (0.5)^b^	196±53 (20)^d^
**D. Bacterial rDNA**	**799F-1391R**	**967F-1391R**	**799F-1193R**	**341F-785R**	**68F-783Rabc**	**68F-518R**	**341F-783Rabc**
Rhizosphere soil	1000±0 (100)^a^	999±0.26 (99)^a^	999±0.3 (99)^a^	998±3 (99)^a^	1000±0 (100)^a^	1000±0 (100)^a^	999±0.52 (99)^a^
Root	1000±0 (100)^a^	414±79 (21)^b^	992±1 (99)^a^	92±41 (9)^b^	250±88 (25)^b^	22±7 (2)^c^	594±72 (60)^d^
Stem	997±3 (99)^a^	2±3 (0.2)^b^	982±11 (98)^a^	4±2 (0.3)^b^	1±1 (0.1)^b^	1±2 (< 0.1)^b^	25±12 (3)^b^
Leaf	1000±0 (100)^a^	93±35 (9)^b^	989±3 (98)^a^	22±15 (2)^b^	85±37 (9)^b^	10±6 (1)^b^	278±25 (28)^c^

(A) Total number of reads (± standard deviation) obtained per plant compartment for each primer pair and average read length (± standard deviation) before and after quality control (QC). Average number of chloroplast (B) and mitochondrial (C) sequences (non-target DNA) obtained from each plant compartment by the selected primer pairs. (D) Amplification of bacterial rDNA reads. Values were normalized to 1000 reads and are averages of three biologically independent replicates ± standard deviation. Values between brackets represent the average percentage (%) of reads. Sequence counts were statistically analyzed using a one-way ANOVA within each plant compartment to compare primer pairs. Differences at the 95% significance level are indicated with lower case letters (P < 0.05).

The standard flowgram format (SFF) files were deposited in the NCBI Sequence Read Archive (SRA) under the Bioproject number PRJNA318176 and BioSample accession numbers SAMN04633889 to SAMN04633970.

### Isolation of intact chloroplasts to extract pure chloroplast DNA

Intact chloroplasts were isolated from (*Populus tremula* × *P. alba*) leaves following a method described by Cortleven et al. (2011). Briefly, fresh leaves (~10 g) were harvested and homogenized in 100 mL ice-cold grinding buffer (2.0 mM NaEDTA; 1.0 mM MgCl_2_; 1.0 mM MnCl_2_; 50.0 mM Hepes/KOH, pH 7.5; 0.33 M sorbitol; 5.0 mM sodium ascorbate) using a Braun MX-32 mixer. The resulting homogenate was filtered through four layers of Miracloth (pore size: 22–25 μm) and centrifuged (1400 g, 5 min). The pellet was resuspended in 1 mL of grinding buffer whereafter the suspension was loaded on a continuous 10–80% Percoll gradient (3% PEG 6000; 1% Ficoll; 1% BSA) and centrifuged (8000 g, 20 min). Finally, intact chloroplasts were collected after centrifugation (lower band), washed twice with five volumes of grinding buffer and stored at −70°C until chloroplast DNA was extracted. DNA was extracted from intact chloroplasts using the Invisorb Spin Plant Mini Kit following the manufacturer's instructions.

### Quantitative real-time PCR

To evaluate the primer efficiency of the selected primer pairs amplifying pure chloroplast DNA (*Populus tremula* × *P. alba*), we tested all primer pairs in a qPCR set-up. From five chloroplast DNA samples, we made a two-fold dilution series (1:2 up to 1:64). qPCR cycling conditions included: initial denaturation at 94°C for 3 min, followed by 35 cycles of denaturation at 94°C for 1 min, annealing at 53°C for 1 min and extension at 72°C during 1 min; a final extension phase was performed at 72°C during 10 min. Finally, a dissociation curve was generated to verify amplification efficiency. Each reaction contained 2 μl of template DNA, 5 μl 2 x Fast SYBR Green Master Mix (Applied Biosystems, Foster City, CA, USA), 0.3 μl forward and reverse primer (0.3 μM each) and 2.4 μl nuclease-free water in a total volume of 10 μl. PCR efficiencies (*E*) were calculated as *E* = (10^−1∕slope^−1) × 100.

### Statistical analysis

Statistical analyses were performed in R 2.15.1 (The R Foundation for Statistical Computing, Vienna, Austria). Normal distributions of the data were checked with the Shapiro–Wilk test and homoscedasticity of variances was analyzed using either Bartlett's or the Fligner–Killeens test. Significant differences in the variance of parameters were evaluated, depending on the distribution of the estimated parameters, either with ANOVA or the Kruskal Wallis Rank Sum Test. *Post hoc* comparisons were conducted by either the Tukey's Honest Significant Differences tests or Pairwise Wilcoxon Rank Sum tests. Poisson corrections were used for abundance data and distributions of ratios were compared with Pearson's Chi-squared tests. Statistical analysis of multivariate data was performed according to the recommendations of Anderson and Willis (2003). Non-metric multi-dimensional scaling (NMDS) was performed using the Vegan 2.0–8 package in R (Oksanen et al., 2013) with 10,000 iterations. The OTU abundance data were square-root transformed (to downweight quantitatively abundant OTUs) and similarities in the bacterial community structures were displayed with non-metric multi-dimensional scaling (NMDS) with Bray-Curtis distances (Bray and Curtis, 1957). Differences between the *a priori* defined groups were evaluated with permutation-based hypothesis tests, namely analysis of similarities (ANOSIM), an analog of univariate ANOVA. Community richness estimators (number of OTUs, Chao1 estimator, ACE estimator, and Bootstrap) and community diversity estimators (Berger-Parker, Shannon, non-parametric Shannon, QStat, Simpson, and Inverse Simpson indices) were calculated in Mothur using 10,000 iterations.

## Results and discussion

After the *in silico* analyses, our final selection included primers 799F and 783Rabc (Chelius and Triplett, 2001; Sakai et al., 2004), containing several mismatches with the chloroplast DNA. Based on primer-to-target 3′ mismatches, overall coverage and taking into account amplicon length, both primers were matched with two primers to produce four primer pairs, respectively 799F-1391R, 799F-1193R, 341F-783Rabc, and 68F-783Rabc. Further, we included primer pair 341F-785R, as described by Klindworth et al. (2013), to evaluate the performance of the mismatch primer sets with the ideal primer pair for 16S rDNA metabarcoding studies with 454 applications Primer sequences and full results of the *in silico* analysis are displayed in Table 1 and Table S1.

### 454 Pyrosequencing

We analyzed 12 samples derived from four different plant compartments (rhizosphere, roots, stems, and leaves) of poplar (*Populus tremula* × *P. alba*) with seven selected bacterial 16S rDNA primer pairs (Table 1). Sequencing of the amplicon libraries generated a total of 799,429 reads with an average of (± standard deviation) 114,204 (± 12013) reads. Quality metrics are displayed in Table 2 with the total amount of reads obtained per plant compartment and the average read length before and after quality checking and trimming (Table 2A).

### Co-amplification of non-target DNA

Firstly, we evaluated the co-amplification of non-target DNA, e.g., chloroplast and mitochondrial DNA, by all primer pairs in the different plant compartments after normalization to 1000 reads (Table 2B,C). In the rhizosphere, some of the selected primer pairs retrieved minute fractions of chloroplast (967F-1391R: <0.1%, 341F-785R: 0.1%, 341F-783Rabc: <0.1%) and mitochondrial (799F-1193R; <0.1%) sequences. Retrieval of plastid and mitochondrial DNA from rhizosphere soil samples is most likely attributable to trace amounts of decaying root, stem or leaf tissue, and the presence of eukaryotic organisms (mitochondrial sequences) in the soil. Furthermore, rhizosphere soil is inevitably contamined with live and dead root cap border cells which have been shown to remain alive after desquamation from the root corpus (Vermeer and McCully, 1982; Hawes et al., 2000; Bulgarelli et al., 2013).

In the plant compartments, we found significant differences in performance of the primer pairs for co-amplification of non-target DNA (Table 2B,C). As expected, interference of non-target DNA was significantly reduced by the primer pairs containing a forward or reverse primer with incorporated mismatches with chloroplast DNA, e.g., primers 799F and 783Rabc (only in combination with 341F). Primer pairs 799F-1391R and 799F-1193R completely eliminated the co-amplification of plastid DNA sequences in the root samples and plastid/chloroplast sequences in the stem and leaf samples. Indeed, primer 799F and variations thereof have been used to minimize chloroplast contamination in plant samples with varying success and mostly resulting in some co-amplification of chloroplast DNA (Sun et al., 2008; Sagaram et al., 2009; Redford et al., 2010; Trivedi et al., 2010; Bragina et al., 2012; Bulgarelli et al., 2012; Bodenhausen et al., 2013; Santhanam et al., 2014; Schlaeppi et al., 2014). However, although primer 783Rabc (Sakai et al., 2004) displayed 3 mismatches with poplar DNA during *in silico* analyses (Table 1), in an experimental set-up it failed to efficiently eliminate chloroplast DNA amplification (Table 2). Primer pair combination 341F-783Rabc performed reasonably well in the root samples (26% of plastid DNA) but higher chloroplast content from stems and leaf samples resulted in significant co-amplification of chloroplast DNA (80% in the stems and 52% in the leafs) (Table 2). Although primer pair 341F-783Rabc performed better than the other primer pairs (without chloroplast mismatches) (Table 2) in reducing the co-amplification of chloroplast DNA, *in silico* analyses portrayed an incorrect image of the primer potential and endorse that the position of the mismatches are crucial for their effectiveness in PCR amplification (Ayyadevara et al., 2000; Klindworth et al., 2013; Lefever et al., 2013). Complete sequence counts for the chloroplast and mitochondrial sequences and statistical differences are presented in Table 2B,C.

Interestingly, we consistently retrieved more chloroplast sequences from the stem samples as compared to the leaf samples for all primer pairs (except 799F-1193R), although not statistically significant for all primer pairs. Although absolute chloroplast DNA concentration is clearly higher in the leaf samples (being the major photosynthetic organ) than the stem samples, the balance between endophytic bacterial DNA and chloroplast DNA seems to be a more crucial factor in the co-amplifcation of chloroplast DNA. Poplar stems are highly lignified and consist of a high proportion of dead cells (xylem vessels) (Boerjan et al., 2003; Vanholme et al., 2010) with low nutrient content (Siebrecht et al., 2003; Danielsen et al., 2013; Morhart et al., 2013) and therefore most likely harbor fewer total bacterial cells than the leaf samples thereby skewing the balance toward the chloroplast DNA.

Finally, we also calculated the number of bacterial 16S rDNA sequences for each primer pair within each plant compartment (Table 2D). These sequence counts of course correlate with the sequence counts of the retrieved non-target DNA from organellar source.

### Primer efficiency for pure chloroplast DNA (poplar)

**Figure 1:**
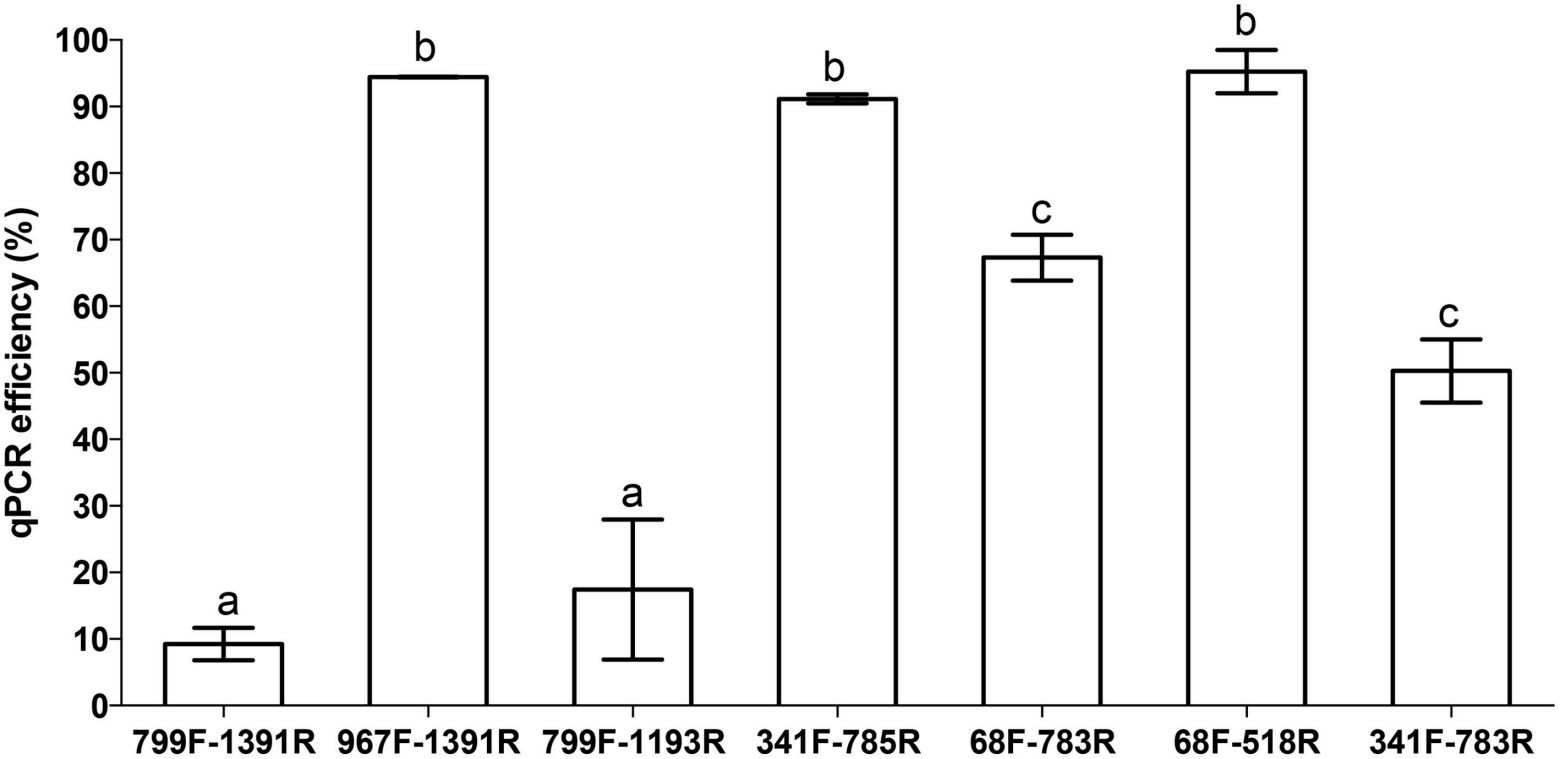
**Average PCR amplification efficiency of selected 16S rDNA primers for pure chloroplast DNA (poplar) using quantitative real-time PCR**. Values are averages of five biologically independent replicates ± standard error. PCR efficiencies were compared using an one-way ANOVA. Differences at the 95% significance level are indicated with lower case letters (*P* < 0.05).

To assess whether or not the observed differences in the amplification of chloroplast sequences in the plant-bacteria DNA extracts during 16S rDNA metabarcoding would correlate with PCR amplification efficiency of the selected primer pairs for pure chloroplast DNA, a qPCR experiment was conducted. To this end, we isolated pure chloroplast DNA from the intact chloroplasts (Cortleven et al., 2011) of 5 poplar leaf samples and amplified the pure chloroplast DNA with the selected primer pairs (Figure 1). We observed a strong correlation between the qPCR set-up and the pyrosequencing results. Primer pairs 799F-1391R and 799F-1193R showed very low affinity for pure poplar chloroplast DNA (efficiency of respectively, 9.2 and 17.4%) resulting in virtually no amplification of chloroplast DNA in the pyrosequencing set-up. Medium affinity for chloroplast 16S DNA was observed for primer pairs 68F-783Rabc (67.3%) and 341F-783Rabc (50.3%) resulting in differential amplification of chloroplast DNA (specifically for 341F-783Rabc) depending on the plastid/chloroplast content (root vs. stem and leaf) in the pyrosequencing set-up. The other primer pairs 967F-1391 (94.5%), 341F-785R (91.1%) and 68F-518R (95.3%) displayed very high PCR amplification efficiencies and indeed resulted in high co-amplification of chloroplast DNA during metabarcoding (Table 2B,C).

### Parametrical comparison of selected primer pairs

**Figure 2:**
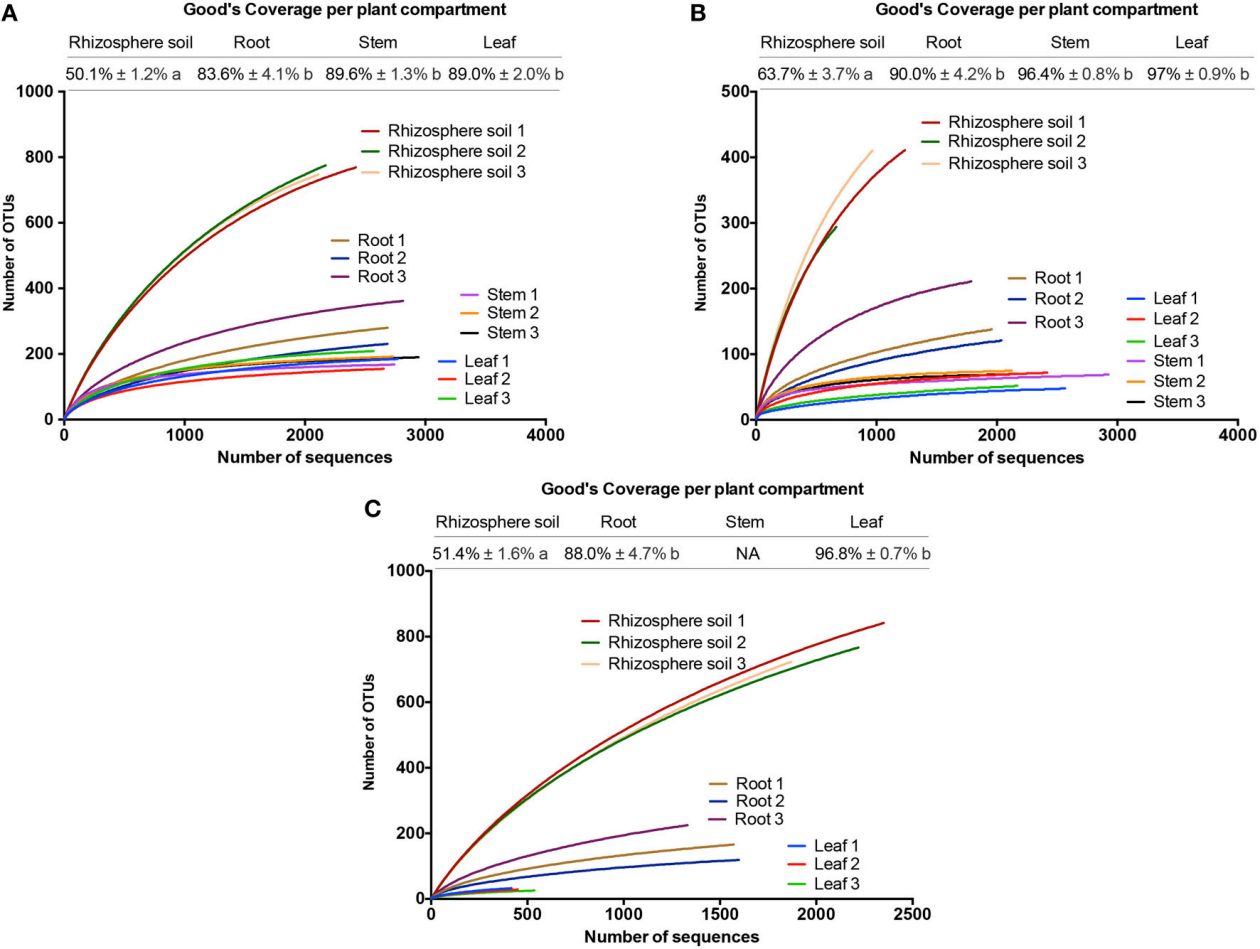
**Good's coverage estimates and rarefaction curves of the different replicates from each plant compartment (rhizosphere soil, root, stem, and leaf) for each primer pair including (A) 799F-1391R, (B) 799F-1193R, and (C) 341F-783Rabc**. Good's coverage estimates were calculated in mothur based on 10,000 iterations. Differences at the 95% significance level between the plant compartments are indicated with lower case letters (*P* < 0.05). Rarefaction curves were assembled showing the number of observed OTUs, defined at a 97% sequence similarity cut-off, relative to the total number of identified bacterial rDNA sequences. To calculate the community estimators, samples were rarified to 417 reads. NA = Not available due to low retrieval of bacterial rDNA reads.

**Figure 3:**
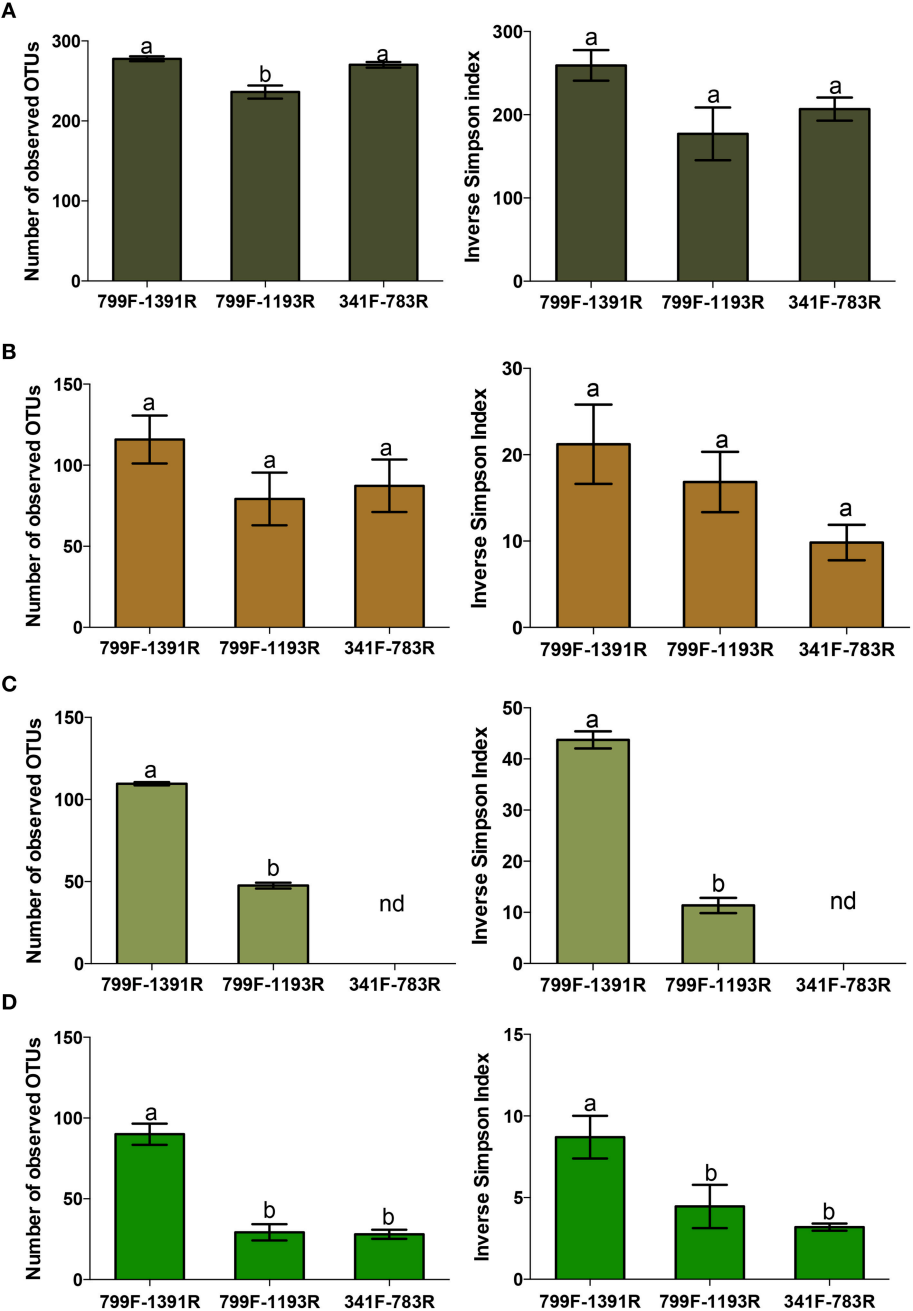
**Comparison of parametrical alpha diversity between selected 16S rDNA primer pairs (799F -1391R, 799F-1193R, and 341F-783Rabc) for all sampled plant compartments [(A) Rhizosphere soil, (B) Root, (C) Stem, (D) Leaf] after subsampling to 417 reads**. All averages were calculated across three biologically independent poplar individuals for each primer pair. Left panels: average number of operational taxonomic units (OTUs) observed based on a 97% sequence similarity cutoff (richness) and right panels: Inverse Simpson diversity indices. OTU counts and Inverse Simpson indices were statistically analyzed using a one-way ANOVA per plant compartment. Differences at the 95% significance level are indicated with lower case letters (*P* < 0.05). nd = not determined as a result of low bacterial rDNA reads.

Based on low co-amplification levels of chloroplast and mitochondrial sequences and consequently high retrieval of bacterial rDNA reads (Table 2D), we selected primer pairs 799F-1391R, 799F-1193R, and 341F-783Rabc for further parametrical analysis (Figures 2, 3).

Rarefaction curves and Good's coverage estimates indicated that our sampling effort (regardless of primer pair) for the rhizospheric samples was inadequate (ranging from 50.1 to 63.7%) to fully capture the bacterial communities (Figure 2). Indeed, rarefaction curves from other studies using rhizosphere soil samples only tend toward saturation after 5000–6000 sequenced reads (Gottel et al., 2011). Observed low Good's coverage estimates were partly the result of our low level of subsampling (417 sequences) used to include as much samples as possible in the parametrical comparison of the primer pairs. Higher subsampling levels (1500 sequences) revealed significantly higher Good's coverage estimates in the rhizosphere soil samples for all primer pairs (ranging from 76 to 91%; data not shown). However, the comparison of the selected primer pairs revealed the highest OTU richness for primer pairs 799F-1391R and 341F-783Rabc, which yielded on average the same amount of OTUs (*P* = 0.80) with 277 OTUs (min = 271; max = 281) and 270 OTUs (min = 265; max = 276), per sample respectively. For primer pair 799F-1193R, OTU richness was significantly lower (*P* < 0.01) with on average 236 OTUs (min = 227; max = 252) per sample (Figure 3A). Inverse Simpson diversity estimates were comparable for all primers (Figure 3A).

For the root, stem and leaf samples, rarefaction curves of all primer pairs tended toward saturation demonstrating that the sequencing effort was sufficient to obtain the most abundant bacterial OTUs. Good's coverage estimates ranged from 83.6% to up to 97% (Figure 2). In the roots, the comparison of the selected primer pairs again revealed the highest OTU richness for primer pair 799F-1391R which obtained on average 115 OTUs (min = 93; max = 143) per sample. This in comparison with primer set 799F-1193R which retrieved 79 OTUs (min = 58; max = 111) per sample and primer pair 341F-783Rabc which yielded 87 OTUs (min = 62; max = 117) (Figure 3B). Gottel et al. (2011) reported high variability in the OTU retrieval isolated from the roots of poplar trees in mature, natural ecosystems (83 OTUs per sample ± 78). However, important to mention, is that we rarefied our samples to 417 sequences per sample, thereby reducing the amounts of OTUs retrieved. Indeed much higher OTUs richness counts were observed in the roots and leaves of *Arabidopsis thaliana* (Bulgarelli et al., 2012; Lundberg et al., 2012; Bodenhausen et al., 2013). Inverse Simpson diversity estimates displayed a clear trend toward higher diversity estimates in primer 799F-1391R as compared to primer pairs 799F-1193R and 341F-783Rabc although not statistically significant for the rhizosphere soil and root samples (Figure 3B).

Finally, in the stem and leaves (Figures 3C,D), we consistently observed the highest OTU richness for primer pair 799F-1391R (*P* < 0.01). In the stem (Figure 3C), primer pair 799F-1391R retrieved on average 109 OTUs (min = 107; max = 110). In contrast, primer set 799F-1193R only obtained 47 OTUs (min = 45; max = 51) per sample in the stems. For primer pair 341F-783Rabc, OTU richness could not be determined in the stem samples (nd) as a consequence of very low amplification of bacterial rDNA reads (Table 2 and Figure 3C). In the leaves (Figure 3D), primer pair 799F-1391R yielded on average 90 OTUs (min = 80; max = 102) whereas primer sets 799F-1193R and 341F-783Rabc retrieved significantly less (*P* < 0.01) OTUs with respectively, 29 OTUs (min = 22; max = 39) and 28 OTUs (min = 23; max = 33) per sample. For the Inverse Simpson index in the stems and leaves, primer pair 799F-1391R consistently showed a higher diversity as compared to primer pairs 799F-1193R and 341F-783Rabc (*P* < 0.01). Diversity indices could not be calculated for primer pair 341F-783Rabc in the stem samples due to low amplification of bacterial reads.

To exclude bias in the community richness and diversity estimators, we included several other alternative estimators (Table S2). Most estimators re-enforce the results obtained from the number of observed OTUs and the inverse Simpson index in Figure 3 and resulted in similar trends, although not all statistically significant.

### Community similarity between primer pairs and plant compartments

To compare the bacterial communities retrieved by each selected primer pair (799F-1391R, 799F-1193R, and 341F-783Rabc) at the phylum and genus level, relative frequency distributions of the obtained genus-level OTUs and phyla were analyzed with chi-squared tests for the three primer pairs, based on average abundances across replicate samples. At the phylum and genus-level, differences were observed (*P* < 0.05) for all three primer pairs within all plant compartments. Further, we compared phylum and genus-level OTU abundances of samples for each primer pair within every plant compartment using non-metric multi-dimensional scaling (NMDS) with Bray-Curtis dissimilarities (Figure S3). The observed dissimilarities in the recovery of OTUs, according to the selected primer pair, at phylum-level (Figure S3a, ANOSIM: *P* < 0.05) and genus-level (Figure S3b, ANOSIM: *P* < 0.05) demonstrate the major bias primer selection can introduce in metabarcoding studies (Aird et al., 2011; Berry et al., 2011; Pinto and Raskin, 2012; Ghyselinck et al., 2013; Klindworth et al., 2013; Op De Beeck et al., 2014; Tremblay et al., 2015).

Finally, we compared the bacterial communities (NMDS) retrieved per plant compartment within each primer pair (Figure S4). In general, irrespective of the primer pair, significantly different OTUs (ANOSIM: *P* < 0.05) were detected in each plant compartment at the phylum level (Figure S4a) and genus-level (Figure S4b) clearly illustrating the specific niche differentiation of plant-associated bacteria. Lower intravariability of the samples (mainly at the phylum level) was observed for primer pair 341F-783R resulting in slightly lower stress values (and slightly higher significance levels) of the NMDS fit as compared to the other primer pairs. However, primer pair 341F-783R also displayed significantly lower OTU richness and diversity index estimates (Figure 3) indicating that lower intravariability of the samples is instigated by low (and less diverse) retrieval of OTUs.

We previously observed the same niche differentiation in the isolation of cultivable bacteria from poplar trees in the same field study (Beckers et al., 2016). Niche differentiation between the rhizosphere and root endophyte microbiome has been described for mature poplar trees growing in natural ecosystems (*Populus deltoides*) (Gottel et al., 2011; Shakya et al., 2013), for *Arabidopsis thaliana* ecotypes (Bulgarelli et al., 2012; Lundberg et al., 2012; Schlaeppi et al., 2014; Bai et al., 2015) and other plant species (Inceoğlu et al., 2010; Weinert et al., 2011; Ofek-Lalzar et al., 2014; Edwards et al., 2015). Recently, Bulgarelli et al. (2013) proposed a two-step selection model for root microbiota differentiation from the rhizosphere where rhizodeposition and host genotype-dependent fine-tuning converge to select specific endophytic assemblages. Although we used a limited amount of biological replicates (3), our data indicate additional fine-tuning and niche differentiation of the microbiota in the aerial plant organs, with the stem and leaf bacterial communities being remarkably dissimilar from the root and rhizosphere (Figure S4). This somewhat in contrast with the findings of Bai et al. (2015) who reported an extensive overlap in taxonomy and genome-encoded functional competences between the leaf and root microbiota of *Arabidopsis thaliana*. Although, some of their evidence from recolonization experiments also pointed to microbiome habitat speciation to their respective ecological niche (Bai et al., 2015).

### Core microbiome members identified by each primer pair

Finally, we also obtained a first look at the rhizospheric and endophytic bacterial communities associated with the different plant compartments of poplar trees (*Populus tremula* × *P. alba*) in the field trial under investigation.

Remarkably, we found a clear difference in the numbers of reads that could not be unambiguously classified at the phylum level in the V6-V7 region (799F-1391R: 46% ± 0.5, 799F-1193R: 49% ± 2.6) as compared to the V3-V4 region (341F-783Rabc: 21% ± 0.5) (*P* < 0.05) in the rhizosphere samples. This is indicative of an insufficient database representation of the biodiversity of soil-borne bacteria and an underrepresentation of the hypervariable V6-V7 region (Gans et al., 2005; Bulgarelli et al., 2012). Indeed, V3-V4 has been the preferred region for next-generation studies (Klindworth et al., 2013). Therefore, at least for the time being, for the study of plant-associated bacteria a trade-off enforces itself to choose for the V6-V7, with the availability of primers to avoid co-amplification of organellar DNA but with an underrepresentation of sequences in this region in the databases.

**Figure 4:**
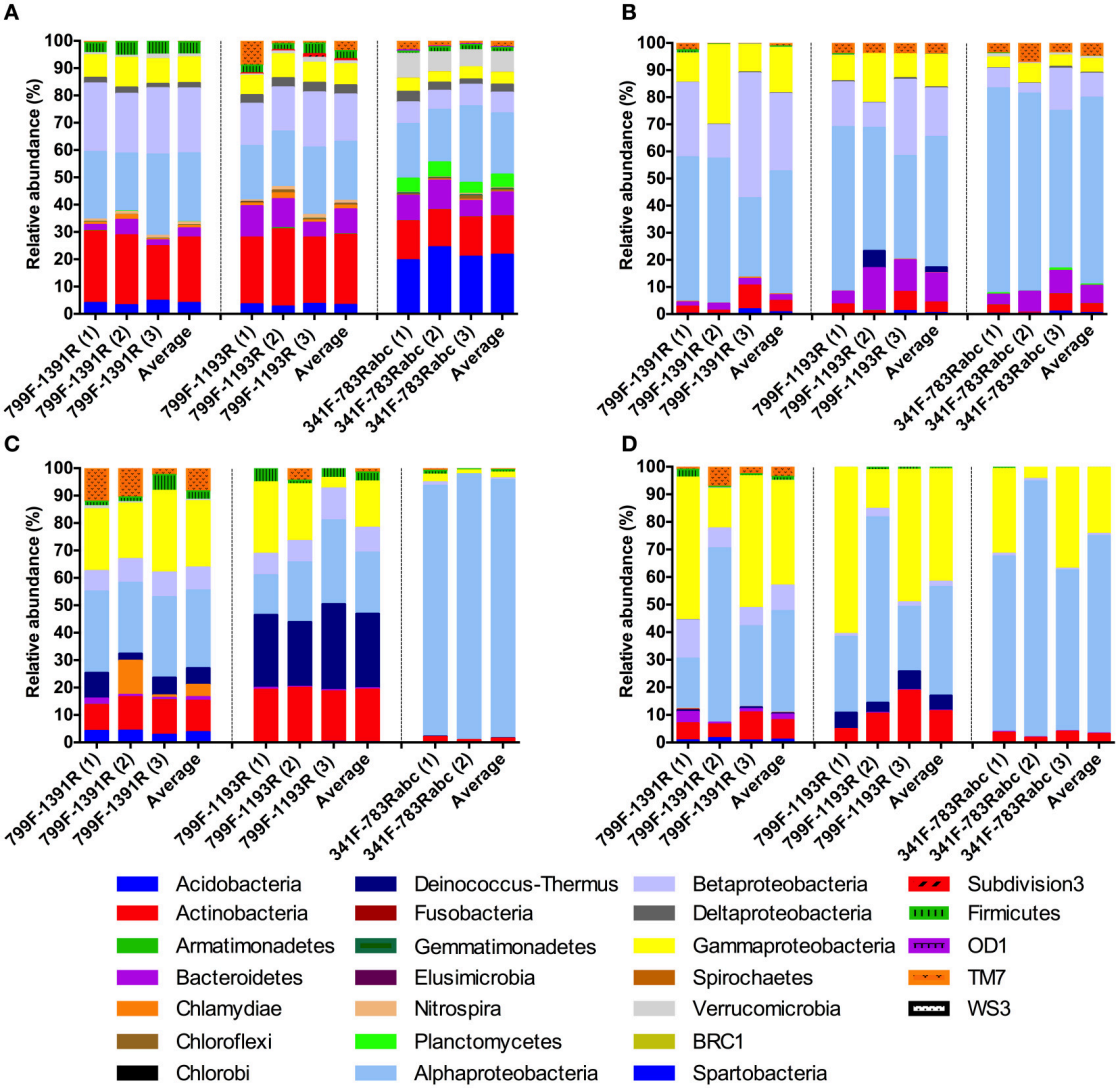
**Relative sequence abundance of bacterial phyla associated with different plant compartments [(A) Rhizosphere soil, (B) Root, (C) Stem, (D) Leaf] identified by the three selected primer pairs (799F-1391R, 799F-1193R, and 341F-783Rabc)**. Proteobacteria OTU has been replaced by 4 OTUs at the subclass level (alpha, beta, gamma, delta). Replicates are displayed in separate bars and also are averaged per primer pair.

At the phylum level (Figure 4), the majority of the OTUs in the rhizosphere soil (Figure 4A) identified by all primer pairs were assigned to Proteobacteria (37 to 60%), Actinobacteria (14 to 25%), Acidobacteria (3 to 21%), and Bacteriodetes (3 to 9%). In the roots (Figure 4B), for all primer pairs, we observed a strong dominance of Proteobacteria (78 to 91%) with a minority of the identified OTUs belonging to Bacteriodetes (2 to 11%), Actinobacteria (2 to 4%), and TM7 (1 to 4%). In the stems (Figure 4C), the dominance of phylum Proteobacteria (48 to 97%) persisted although it was slightly less pronounced for the analyses on primer pairs 799F-1391R (61%) and 799F-1193R (48%) compared to primer pair 341F-783Rabc (97%). Most likely, this observation is directly related to the very reduced number of bacterial sequences obtained from the stem samples by primer pair 341F-783Rabc. For the analyses based on primer pairs 799F-1391R and 799F-1193R, the rest of the identified OTUs mainly belonged to Actinobacteria (respectively 11 and 19%) and Deinococcus-Thermus (respectively 6 and 27%). Finally in the leaves, the majority of the OTUs were also identified as Proteobacteria (82 to 96%). A minority of the OTUs found in the leaves belonged to Actinobacteria lineages (3 to 11%) (Figure 4D).

Taking a closer look at the phylum level, in the rhizosphere we predominantly identified *Proteobacteria, Acidobacteria*, and *Actinobacteria*, irrespective of the selected primer pair. The ratio of *Proteobacteria* to *Acidobacteria* in rhizosphere bacterial communities has previously been shown to be an indicator of soil nutrient content where *Proteobacteria* were linked to nutrient-rich soils and *Acidobacteria* to nutrient-poor soils (Smit et al., 2001; Castro et al., 2010; Gottel et al., 2011). Endophytic communities were, for the most part dominated by *Proteobacteria* suggesting substantial overlap in key community members across host species (Gottel et al., 2011; Bulgarelli et al., 2012; Lundberg et al., 2012; Bodenhausen et al., 2013; Shakya et al., 2013; Romero et al., 2014).

To give an idea of the bacterial communities at the genus level, we defined the core bacterial community, described by each primer pair, as the 10 most abundant genus-level OTUs per compartment. This resulted in 21 OTUs for the rhizosphere, 18 OTUs for the roots, and 23 OTUs for the stem and leaf samples. The percentage of sequences represented by these 10 most abundant OTUs for each primer pair and plant compartment are listed in Tables S3–S6. For all plant compartments, and in particular the rhizosphere soil, we observed long-tailed rank-abundance curves characteristic of microbial communities (Hartmann et al., 2012). Worth noting here is that for all compartments, except for the leaves, the most abundantly identified OTU was different for each primer pair. This demonstrates the major effect of primer choice on the observed bacterial communities. In the rhizosphere, *Actinomycetales* (9.9%, 799F-1391R), *Rhizobiales* (11.3%, 799F-1193R), and *Acidobacteria_Gp6* (15.7, 341F-783Rabc) were the dominant OTUs. In the roots, *Pseudomonas* (11.9%, 799F-1391R), *Rhizobium* (15.9%, 799F-1193R), and *Rhizobiales* (38.9%, 341F-783Rabc) constituted the major identified OTUs. In the stems, *Pseudomonas* (12.9%, 799F-1391R), *Deinococcaceae* (18.2%, 799F-1193R), and *Sphingomonadaceae* (34.2%, 341F-783Rabc) were the most observed OTUs. Finally, in the leaves, *Pseudomonas* dominated bacterial communities regardless of the primer pair used.

At genus-level, most remarkable is the efficiency of the endophytic colonization of the OTU *Pseudomonas* as indicated by all studied primer pairs (Tables S3–S6). A low relative abundance of *Pseudomonas* in the rhizosphere soil (0.3 to 1.9%) is contrasted by its dominance in the endosphere samples (3.67 to 40.13%). Similarly, comparative studies revealed high relative abundance of a *Pseudomonas*-like OTU in the root microbiome of *Populus deltoides* (Gottel et al., 2011), although a new study revealed similar dominance in the root endosphere by a *Streptomyces*-like OTU (Shakya et al., 2013). In contrast, root and leaf microbiota of *Arabidopsis* genotypes appear to be not heavily populated by *Pseudomonas*-like OTUs (Bulgarelli et al., 2012; Lundberg et al., 2012; Schlaeppi et al., 2014; Bai et al., 2015). Endophytic colonization of *Pseudomonas* may occur via the rhizosphere where most endophytic bacteria should originate from and/or via leaf stomatal colonization since aerosol samples were found to harbor abundant *Pseudomonas* sequences (Fahlgren et al., 2010). Finally, important to mention with the interpretation of community studies with next generation sequencing platforms is the 16S rRNA operon copy number. This copy number may vary, depending on species, from 1 to 15, introduces significant bias and distort views on bacterial communities (Crosby and Criddle, 2003; Lee et al., 2009). Furthermore, to fully evaluate the reliability and robustness of the primers concerning bias against and/or in favor of specific taxonomic groups and community richness and diversity estimators, the use of mock communities would provide more insight into their experimental behavior during metabarcoding studies (Caporaso et al., 2011; Pinto and Raskin, 2012).

## Concluding remarks

We experimentally evaluated the performance of seven 16S rDNA primers pairs in 16S rDNA metabarcoding studies of endophytic and rhizospheric bacterial communities. Our results show that different primer pairs display different efficiencies in the elimination of non-target DNA. In this study, the primer pair 799F-1391R, which amplifies the V5-V7 hypervariable regions of the 16S rDNA gene, displayed very low amplification of non-target DNA across all sampled plant compartments. And retrieved the highest number of OTUs as well as exhibited the highest Inverse Simpson diversity, especially in the plant compartments with high chloroplast content (stem and leaf samples). Therefore, we propose primer pair 799F-1391R as best suited for 16S rDNA metabarcoding studies which simultaneously investigate rhizosphere and endosphere microbiomes. Specifically, in experimental set-ups where direct comparisons are made between different endosphere microbiomes such as the microbiomes of wild-type plants and genetically modified plants. However, to perform an in-depth characterization of the rhizosphere and/or endosphere microbiomes and uncover the true bacterial diversity, the use of multiple primer pairs is highly advisable.

Recently, the use of other 16S rDNA metabarcoding applications such as the HiSeq2000, MiSeq Illumina and Ion Torrent platforms have come to the foreground (Claesson et al., 2010; Metzker, 2010; Caporaso et al., 2012; Logares et al., 2013; Kennedy et al., 2014). However, in plant-microbiota research, the application of these platforms has been mainly limited, up till now, to a couple of studies of the rhizosphere microbiome (Jiang et al., 2013; Panke-Buisse et al., 2014; Sun et al., 2014) and root and leaf microbiota (Ofek-Lalzar et al., 2014; Bai et al., 2015; Coleman-Derr et al., 2016). Evaluating the potential of our optimized approach, with platform-specific modifications (e.g., amplicon length), in combination with HiSeq2000 and MiSeq Illumina could further contribute to the high-resolution 16S rDNA-based community profiling of plant-associated bacterial communities.

## Author contributions

Conceptualization: BB, JV, and WB; Methodology: BB, MODB, SoT, and SaT; Investigation: BB; Writing—original draft: BB; Writing—Review and editing: MODB, NW, WB, and JV; Funding Acquisition: JV and WB.

## Funding

This work was funded by the Fund for Scientific Research Flanders (FWO-Vlaanderen), project number G032912N, Ph.D. grants for Michiel Op De Beeck, Sofie Thijs and Sascha Truyens and a post-doc grant for Nele Weyens. Furthermore, this work has been financially supported by the UHasselt Methusalem project 08M03VGRJ.

## Data accessibility

The standard flowgram format (SFF) files were deposited in the NCBI Sequence Read Archive (SRA) under the Bioproject number PRJNA318176 and BioSample accession numbers SAMN04633889 to SAMN04633970.

### Conflict of interest statement

The authors declare that the research was conducted in the absence of any commercial or financial relationships that could be construed as a potential conflict of interest.
