# Supplementary material for: Performance of 16s rDNA Primer Pairs in the Study of Rhizosphere and Endosphere Bacterial Microbiomes in Metabarcoding Studies
Source: Front Microbiol. 2016 May 13;7:650. doi: 10.3389/fmicb.2016.00650 (PMC4865482; doi:10.3389/fmicb.2016.00650)
Supplement: Supplementary file 1 [file Image1.PDF]

Supplementary Material (Beckers et al., 2016)

Supplementary Figures

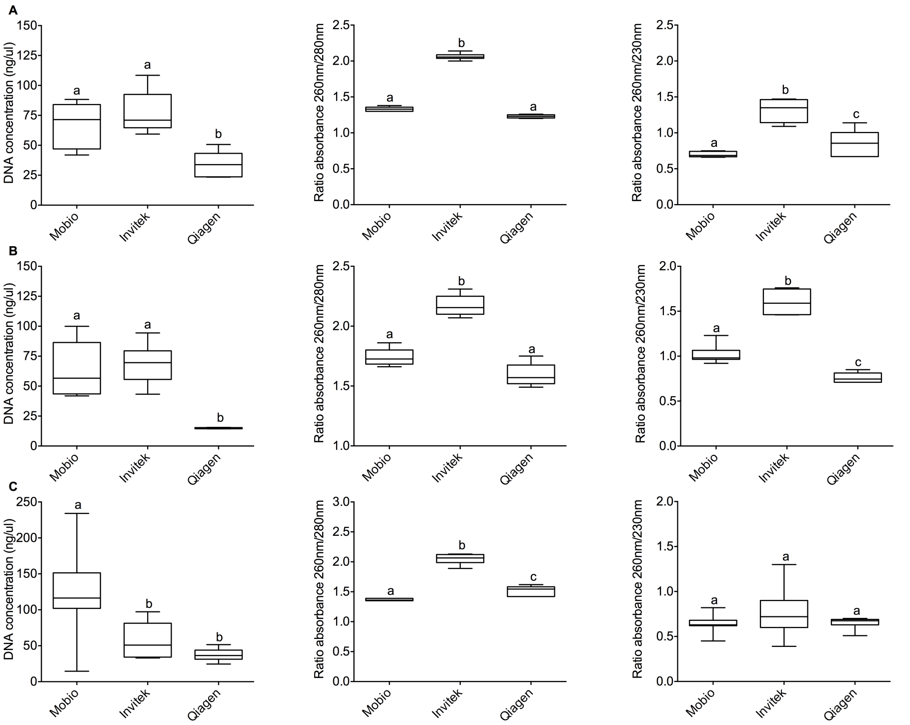

Figure S1 <u>Performance of three different commercially available DNA extraction kits (Mobio Powerplant DNA Isolation Kit, Invitek Invisorb Spin Plant Mini Kit and the Qiagen DNeasy Plant Mini Kit).</u> DNA was extracted from roots (A), stems (B) and leafs (C) of poplar trees (*Populus tremula x alba*). Quantity and quality (absorbance ratios of 260nm/280 and 230nm/260nm) of extracted DNA was evaluated for each kit using a Nanodrop ND-1000 Spectrophotometer. Differences at the 95% confidence interval are displayed with lower cased letters ($P < 0.05$).

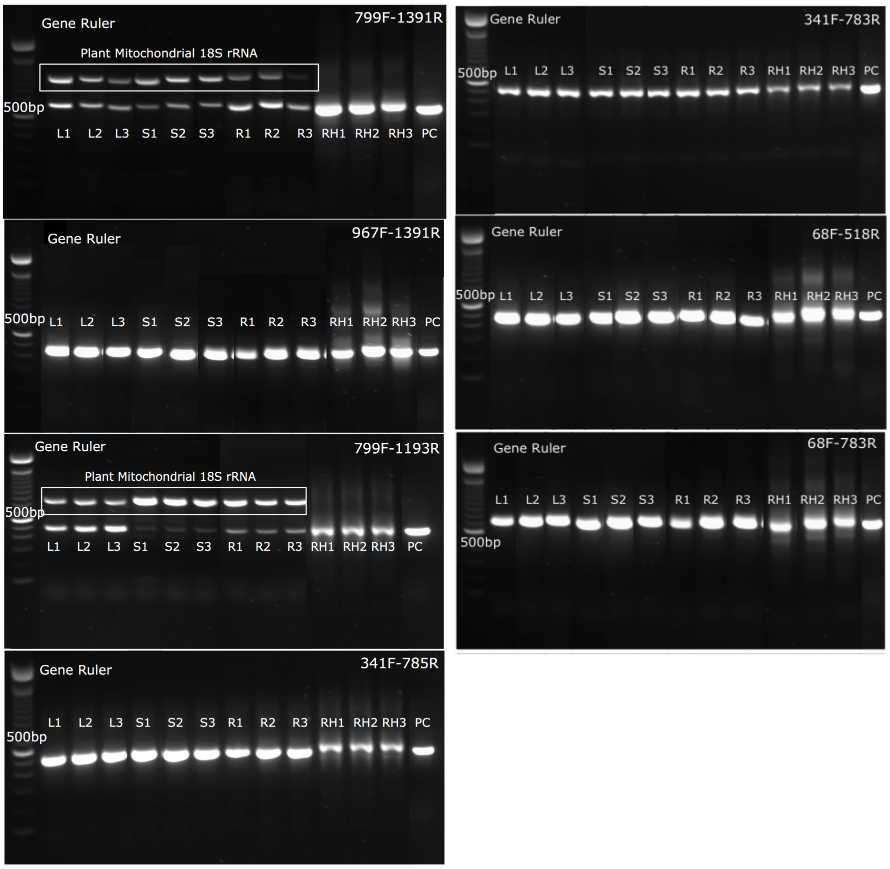

Figure S2: Agarose gels (1.5%) of amplicons produced with all selected primer pairs from the different plant compartments (L = Leaf, S = Stem, R = Root, RH = Rhizosphere soil). Gels were run for 2.5 hours at 90 V and illuminated using UV-light. Positive control (PC) was the PCR product with as template pure bacterial DNA from a cultured bacterial strain (*Pseudomonas putida*). To check amplicon sizes, a 1kB gene ruler was used (band of 500 bp indicated).

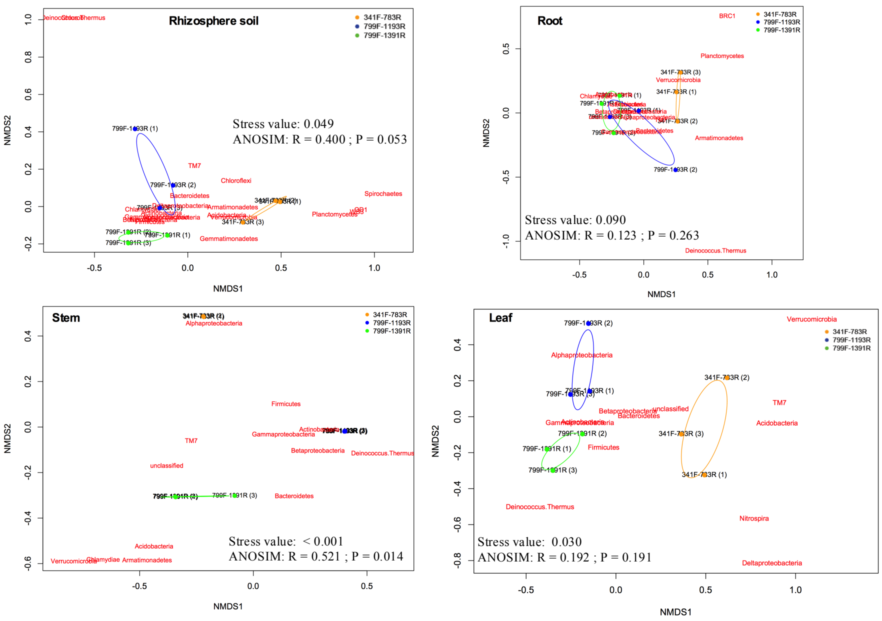

Figure S3a Observed dissimilarities in the phylum-level OTU abundances identified by the selected primer pairs (799F-1391R, 799F-1193R and 341F-783R) within every plant compartment using non-metric multi-dimensional scaling (NMDS) with Bray-Curtis dissimilarities (10,000 permutations). Bacterial phyla are displayed in red. Stress values and results from ANOSIM statistical analysis are displayed within each individual NMDS plot. Ellipses superimposed on the NMDS plots were calculated using the 'Vegan Package' in R, function ordiellipse and were based on the standard deviation of the OTU data.

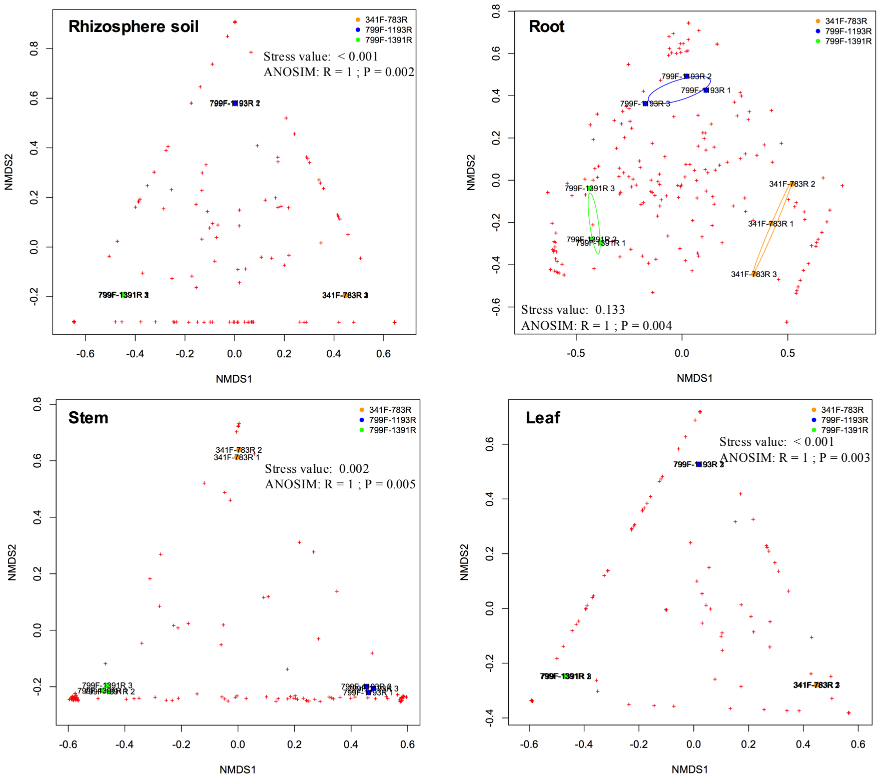

Figure S3b Observed dissimilarities in the genus-level OTU abundances identified by the selected primer pairs (799F-1391R, 799F-1193R and 341F-783R) within every plant compartment using non-metric multi-dimensional scaling (NMDS) with Bray-Curtis dissimilarities (10,000 permutations). Bacterial genera are displayed in red. Stress values and results from ANOSIM statistical analysis are displayed within each individual NMDS plot. Ellipses superimposed on the NMDS plots were calculated using the 'Vegan Package' in R, function ordiellipse and were based on the standard deviation of the OTU data.

a

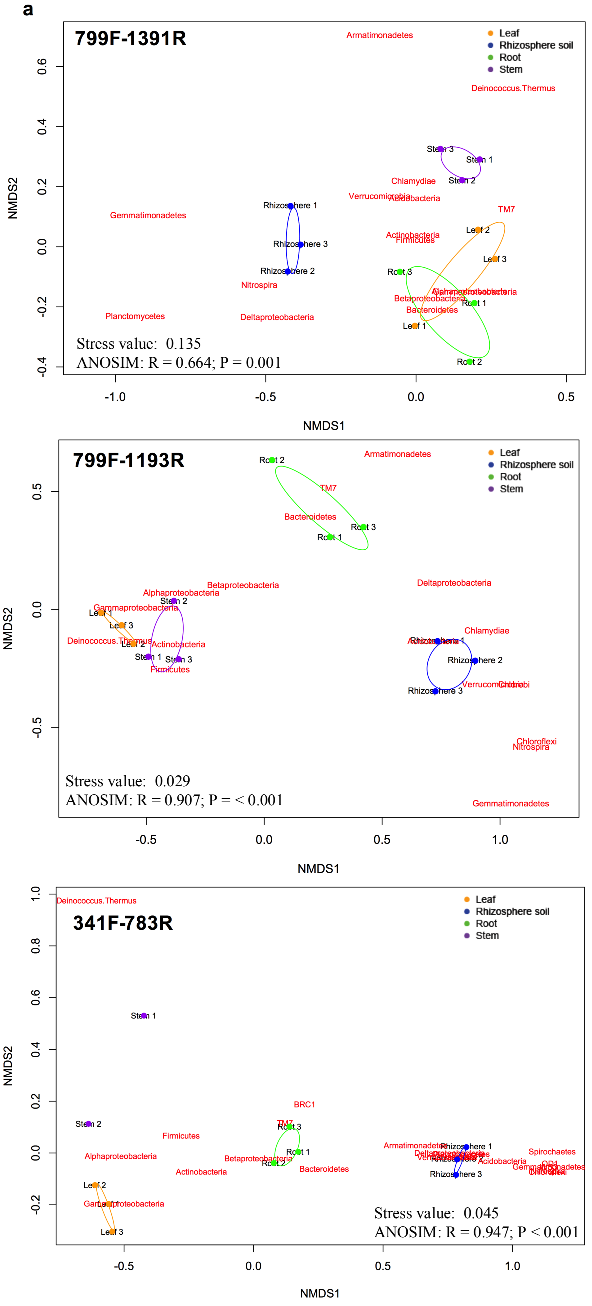
799F-1391R
Leaf
Rhizosphere soil
Root
Stem
Armatimonadetes
Deinococcus.Thermus
Gemmatimonadetes
Planctomycetes
Nitrospira
Deltaproteobacteria
Chlamydiae
Verrucomicrobia
Acidobacteria
TM7
Actinobacteria
Firmicutes
Betaproteobacteria
Bacteroidetes
Stress value: 0.135
ANOSIM: R = 0.664; P = 0.001
NMDS1
NMDS2
799F-1193R
Armatimonadetes
TM7
Bacteroidetes
Betaproteobacteria
Alphaproteobacteria
Gammaproteobacteria
Deinococcus.Thermus
Actinobacteria
Firmicutes
Deltaproteobacteria
Chlamydiae
Chloroflexi
Nitrospira
Gemmatimonadetes
Stress value: 0.029
ANOSIM: R = 0.907; P = < 0.001
NMDS1
NMDS2
341F-783R
Deinococcus.Thermus
BRC1
TM7
Firmicutes
Alphaproteobacteria
Actinobacteria
Betaproteobacteria
Bacteroidetes
Armatimonadetes
Acidobacteria
Spirochaetes
OD1
Stress value: 0.045
ANOSIM: R = 0.947; P < 0.001
NMDS1
NMDS2

Figure S4a Observed dissimilarities in the phylum-level OTU abundances in each plant compartment (rhizosphere soil, root, stem and leaf) by the selected primer pairs (799F-1391R, 799F-1193R and 341F-783R) using non-metric multi-dimensional scaling (NMDS) with Bray-Curtis dissimilarities (10,000 permutations). Bacterial phyla are displayed in red. Stress values and results from ANOSIM statistical analysis are displayed within each individual NMDS plot. Ellipses superimposed on the NMDS plots were calculated using the 'Vegan Package' in R, function ordiellipse and were based on the standard deviation of the OTU data.

b

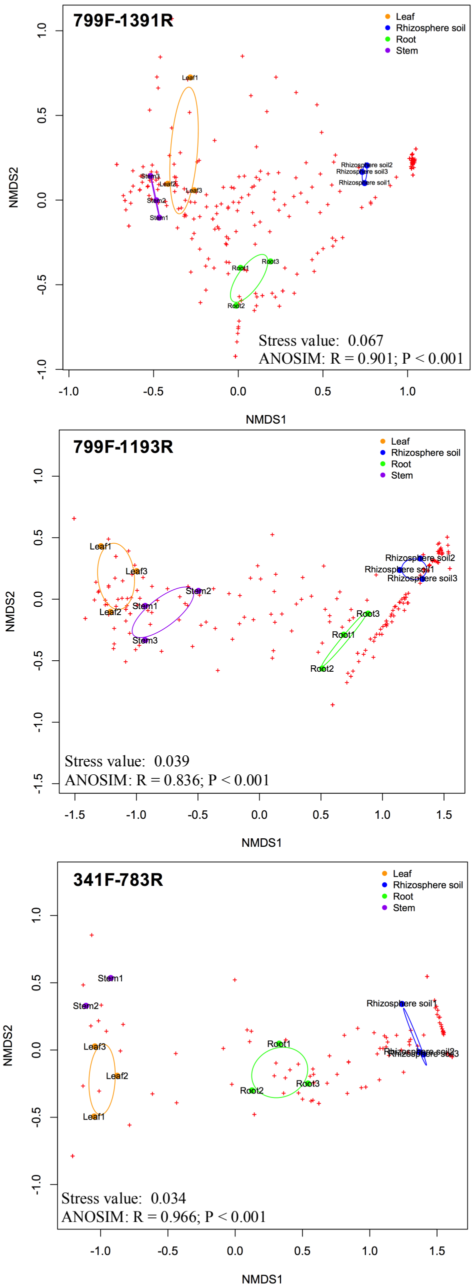
799F-1391R
Leaf
Rhizosphere soil
Root
Stem
Stress value: 0.067
ANOSIM: R = 0.901; P < 0.001
NMDS1
NMDS2
799F-1193R
Leaf
Rhizosphere soil
Root
Stem
Stress value: 0.039
ANOSIM: R = 0.836; P < 0.001
NMDS1
NMDS2
341F-783R
Leaf
Rhizosphere soil
Root
Stem
Stress value: 0.034
ANOSIM: R = 0.966; P < 0.001
NMDS1
NMDS2

Figure S4b Observed dissimilarities in the genus-level OTU abundances in each plant compartment (rhizosphere soil, root, stem and leaf) by the selected primer pairs (799F-1391R, 799F-1193R and 341F-783R) using non-metric multi-dimensional scaling (NMDS) with Bray-Curtis dissimilarities (10,000 permutations). Bacterial genera are displayed in red. Stress values and results from ANOSIM statistical analysis are displayed within each individual NMDS plot. Ellipses superimposed on the NMDS plots were calculated using the 'Vegan Package' in R, function ordiellipse and were based on the standard deviation of the OTU data.

Supplementary tables

Table S1: Primer Prospector analysis

**GreenGenes Database gg_13_5**		1,262,986 sequences	
**Curated GreenGenes database**		946,815 sequences	
**Primer**	**Number of 3' mismatches**	**% 3' mismatches**	**Primer Score**
799F	37795	3.99	0.29
1391R	42099	4.45	1.44
967F	1926	0.20	0.34
1391R	42099	4.45	1.44
799F	37795	3.99	0.29
1193R	3444	0.36	0.2
341F	3536	0.37	0.05
785R	3464	0.37	0.09
68F	3536	0.37	0.60
783Ra	180197	19.03	4.60
783Rb	466397	49.26	5.17
783Rc	394841	41.70	5.38
68F	26040	2.75	0.60
518R	9309	0.98	0.09
341F	26040	2.75	0.05
783Ra	180197	19.03	4.60
783Rb	466397	49.26	5.17
783Rc	394841	41.70	5.38

Primer scores were calculated based on the following formula: weighted score =non-3' mismatches x 0.40 + 3' mismatches x 1.00 + non-3' gaps x 1.00 + 3' gaps x 3.00. An additional penalty score of 3.00 was assigned if the final 3' base of a primer had a mismatch with its target sequence (Walters et al., 2011)

Table S2: Alternative community richness and diversity estimators

Chao

Primerpair	Rhizosphere soil	Root	Stem	Leaf
799F-1391R	785.10 ± 21.05 a	242.61 ± 60.22 a	154.29 ± 11.89 a	155.66 ± 26.09 a
799F-1193R	470.28 ± 64.91 b	148.29 ± 52.74 a	62.93 ± 7.54 b	48.64 ± 12.27 b
341F-783Rabc	788.18 ± 43.76 a	185.05 ± 69.28 a	NA	46.68 ± 6.76 b

ACE

Primerpair	Rhizosphere soil	Root	Stem	Leaf
799F-1391R	1345.45 ± 148.00 a	375.61 ± 117.02 a	167.38 ± 26.17 a	202.40 ± 42.59 a
799F-1193R	610.30 ± 124.75 b	232.92 ± 100.34 a	69.46 ± 13.81 b	70.35 ± 14.15 b
341F-783Rabc	1360.62 ± 146.37 a	265.46 ± 106.10 a	NA	68.88 ± 21.49 b

Bootstrap

Primerpair	Rhizosphere soil	Root	Stem	Leaf
799F-1391R	360.62 ± 7.23 a	143.95 ± 32.55 a	128.99 ± 3.68 a	109.57 ± 14.82 a
799F-1193R	299.48 ± 19.45 b	96.54 ± 35.53 a	54.39 ± 3.95 b	34.56 ± 10.38 b
341F-783Rabc	350.81 ± 8.38 a	107.88 ± 36.00 a	NA	33.60 ± 6.08 b

Berger-Parker

Primerpair	Rhizosphere soil	Root	Stem	Leaf
799F-1391R	0.03 ± 0.01 a	0.16 ± 0.03 a	0.08 ± 0.01 a	0.31 ± 0.08 a
799F-1193R	0.04 ± 0.01 a	0.15 ± 0.02 a	0.21 ± 0.06 b	0.46 ± 0.16 a
341F-783Rabc	0.04 ± 0.003 a	0.27 ± 0.03 b	NA	0.51 ± 0.02 a

Shannon

Primerpair	Rhizosphere soil	Root	Stem	Leaf
799F-1391R	5.41 ± 0.04 a	3.79 ± 0.39 a	4.16 ± 0.02 a	3.24 ± 0.02 a
799F-1193R	5.19 ± 0.11 a	3.36 ± 0.42 a	2.94 ± 0.15 b	1.99 ± 0.44 b
341F-783Rabc	5.34 ± 0.04 a	3.17 ± 0.46 a	NA	1.73 ± 0.21 b

np Shannon

Primerpair	Rhizosphere soil	Root	Stem	Leaf
799F-1391R	6.11 ± 0.06 a	4.09 ± 0.42 a	4.36 ± 0.02 a	3.51 ± 0.07 a
799F-1193R	5.71 ± 0.15 a	3.56 ± 0.48 a	3.04 ± 0.15 b	2.09 ± 0.46 b
341F-783Rabc	6.01 ± 0.07 a	3.43 ± 0.53 a	NA	1.84 ± 0.22 b

Q Stat

Primerpair	Rhizosphere soil	Root	Stem	Leaf
799F-1391R	180.89 ± 4.27 a	58.08 ± 16.86 a	42.49 ± 0.73 a	38.98 ± 7.82 a
799F-1193R	145.09 ± 10.90 b	31.22 ± 18.45 a	14.12 ± 0.52 b	7.56 ± 4.14 b
341F-783Rabc	175.07 ± 5.27 a	39.40 ± 19.23 a	NA	8.51 ± 1.79 b

Simpson

Primerpair	Rhizosphere soil	Root	Stem	Leaf
799F-1391R	0.004 ± 0.0005 a	0.05 ± 0.02 a	0.02 ± 0.001 a	0.12 ± 0.03 a
799F-1193R	0.006 ± 0.002 a	0.06 ± 0.02 a	0.09 ± 0.02 b	0.26 ± 0.12 a,b
341F-783Rabc	0.005 ± 0.0006 a	0.11 ± 0.03 a	NA	0.32 ± 0.04 b

Community richness estimators (Chao1 estimator, ACE estimator and bootstrap estimator) and community diversity estimators (Berger-Parker Index, Shannon Index, non-parametric Shannon Index, Q statistic and Simpson index) were calculated in Mothur using 10,000 iterations. Lower cased letters indicate significant differences at the 95% confidence interval ($P < 0.05$).

Table S3: Rhizosphere core bacterial community (10 most abundant OTUs, relative abundance %) identified by each primer pair. Most abundant OTU is indicated in bold. OTUs are indicated with the lowest identified taxonomic rank corresponding to the genus-level or higher levels (family, order, class, phylum).

Genus (or higher)	Phylum	799F-1391R	799F-1193R	341F-783R
Rhizobiales	Alphaproteobacteria	1.77	**11.32**	8.36
Actinomycetales	Actinobacteria	**9.96**	8.64	7.73
Burkholderiales	Betaproteobacteria	5.97	2.97	1.68
Variovorax	Betaproteobacteria	4.33	0.00	0.11
Bacillales	Fimicutes	3.85	0.51	0.00
Chitinophagaceae	Bacteriodetes	2.65	2.23	5.65
Bradyrhizobium	Alphaproteobacteria	2.90	1.03	0.29
Arthrobacter	Actinobacteria	2.56	0.91	0.00
Pseudomonas	Gammaproteobacteria	1.93	1.11	0.33
Xanthomonadaceae	Gammaproteobacteria	1.79	1.78	0.77
Flavobacterium	Bacteriodetes	0.23	5.45	0.98
TM7	TM7	0.48	5.55	2.52
Comamonadaceae	Betaproteobacteria	1.13	4.48	1.91
Myxococcales	Deltaproteobacteria	1.03	2.12	2.27
Sphingomonadaceae	Alphaproteobacteria	0.88	2.50	2.06
Ilumatobacter	Actinobacteria	0.00	2.16	0.00
Acidobacteria_Gp6	Acidobacteria	0.00	0.00	**15.73**
Spartobacteria	Verrucomicrobia	0.06	0.00	6.93
Planctomycetaceae	Planctomycetes	0.00	0.00	4.74
Bradyrhizobiaceae	Alphaproteobacteria	0.80	1.52	3.65
Acidobacteria_Gp4	Acidobacteria	0.03	0.00	3.64
**Total amount of reads covered by top 10 OTUs (%)**		42.34	54.25	69.35

Table S4: Root core bacterial community (10 most abundant OTUs, relative abundance %) identified by each primer pair. Most abundant OTU is indicated in bold. OTUs are indicated with the lowest identified taxonomic rank corresponding to the genus-level or higher levels (family, order, class, phylum).

Genus (or higher)	Phylum	799F-1391R	799F-1193R	341F-783R
Pseudomonas	Gammaproteobacteria	**11.95**	9.46	3.67
Rhizobiales	Alphaproteobacteria	11.28	7.39	**38.97**
Variovorax	Betaproteobacteria	8.28	0.00	1.48
Rhizobium	Alphaproteobacteria	6.70	**15.88**	0.00
Afipia	Alphaproteobacteria	6.13	0.00	0.00
Phenylobacterium	Alphaproteobacteria	5.88	5.25	0.22
Burkholderiaceae	Betaproteobacteria	5.52	3.95	1.48
Novosphingobium	Alphaproteobacteria	5.44	4.77	0.00
Burkholderia	Betaproteobacteria	4.72	2.14	0.62
Burkholderiales	Betaproteobacteria	3.34	1.32	2.84
Flavobacterium	Bacteriodetes	0.77	8.38	3.78
Bradyrhizobiaceae	Alphaproteobacteria	1.12	5.57	2.62
Comamonadaceae	Betaproteobacteria	1.20	4.35	1.15
TM7	TM7	1.26	4.27	6.33
Erythrobacteraceae	Alphaproteobacteria	0.00	0.00	5.19
Caulobacteraceae	Alphaproteobacteria	0.17	0.68	4.85
Sphingomonadaceae	Alphaproteobacteria	1.04	1.45	3.37
Oxalobacteraceae	Betaproteobacteria	2.00	3.01	2.59
**Total amount of reads covered by top 10 OTUs (%)**		76.81	77.86	79.17

Table S5: Stem core bacterial community (10 most abundant OTUs, relative abundance %) identified by each primer pair. Most abundant OTU is indicated in bold. OTUs are indicated with the lowest identified taxonomic rank corresponding to the genus-level or higher levels (family, order, class, phylum).

Genus (or higher)	Phylum	799F-1391R	799F-1193R	341F-783R
Pseudomonas	Gammaproteobacteria	**12.94**	12.73	4.86
TM7	TM7	9.83	1.51	4.17
Deinococcus	Deinococcus-Thermus	7.18	5.52	0.69
Xanthomonadaceae	Gammaproteobacteria	4.70	0.14	0.00
Chlamydiales	Chlamydiae	4.49	0.00	0.00
Bradyrhizobiaceae	Alphaproteobacteria	4.46	0.11	0.00
Rhizobiales	Alphaproteobacteria	3.83	1.49	9.28
Propionibacterium	Actinobacteria	3.73	5.93	0.00
Methylobacterium	Alphaproteobacteria	3.65	5.44	1.39
Acidobacteria_Gp1	Acidobacteria	2.59	0.00	0.00
Deinococcaceae	Deinococcus-Thermus	0.00	**18.22**	0.00
Sphingomonas	Alphaproteobacteria	2.44	9.87	0.00
Truepera	Deinococcus-Thermus	0.00	4.55	0.00
Curtobacterium	Actinobacteria	1.17	3.89	0.00
Herbaspirillum	Betaproteobacteria	0.15	3.24	0.00
Enterobacteriaceae	Gammaproteobacteria	1.29	2.72	0.00
Sphingomonadaceae	Alphaproteobacteria	1.21	0.20	**34.28**
Enhydrobacter	Gammaproteobacteria	1.40	0.05	5.56
Staphylococcaceae	Firmicutes	0.08	0.28	5.56
Staphylococcus	Firmicutes	0.95	0.56	5.56
Alishewanella	Gammaproteobacteria	0.00	0.00	4.17
Actinomycetales	Actinobacteria	0.00	1.02	3.47
Microbacteriaceae	Actinobacteria	0.70	1.01	3.47
**Total amount of reads covered by top 10 OTUs (%)**		66.78	78.46	82.44

Table S6: Leaf core bacterial community (10 most abundant OTUs, relative abundance %) identified by each primer pair. Most abundant OTU is indicated in bold. OTUs are indicated with the lowest identified taxonomic rank corresponding to the genus-level or higher levels (family, order, class, phylum).

Genus (or higher)	Phylum	799F-1391R	799F-1193R	341F-783R
Pseudomonas	Gammaproteobacteria	**33.95**	**40.13**	**35.97**
Rhizobium	Alphaproteobacteria	8.45	0.51	0.00
Methylobacterium	Alphaproteobacteria	6.68	6.21	3.99
Rhizobiales	Alphaproteobacteria	5.47	13.65	28.77
TM7	TM7	4.79	0.00	0.00
Sphingomonas	Alphaproteobacteria	3.34	7.20	0.00
Pasteurella	Gammaproteobacteria	2.18	0.00	0.00
Burkholderiales	Betaproteobacteria	2.94	0.01	0.08
Xanthomonadaceae	Gammaproteobacteria	1.73	0.00	0.07
Actinomycetales	Actinobacteria	1.59	0.39	0.14
Rhizobiaceae	Alphaproteobacteria	0.00	6.76	0.00
Microbacteriaceae	Actinobacteria	0.37	5.43	1.26
Deinococcaceae	Deinococcus-Thermus	0.00	4.32	0.00
Aurantimonas	Alphaproteobacteria	0.47	4.26	0.00
Propionibacterium	Actinobacteria	1.37	1.67	0.00
Deinococcus	Deinococcus-Thermus	0.43	1.25	0.00
Sphingomonadaceae	Alphaproteobacteria	0.19	0.05	19.85
Clavibacter	Actinobacteria	1.22	1.02	1.89
Enhydrobacter	Gammaproteobacteria	0.42	0.01	1.13
Pseudomonadaceae	Gammaproteobacteria	0.45	0.00	0.89
Dermacoccus	Actinobacteria	0.31	0.04	0.72
Oxalobacteraceae	Betaproteobacteria	0.29	0.55	0.69
**Total amount of reads covered by top 10 OTUs (%)**		**76.66**	**93.48**	**95.46**
